# Accumulation of Non-Pathological Liver Fat Is Associated with the Loss of Glyoxalase I Activity in Humans

**DOI:** 10.3390/metabo14040209

**Published:** 2024-04-07

**Authors:** Andreas Peter, Erwin Schleicher, Elisabeth Kliemank, Julia Szendroedi, Alfred Königsrainer, Hans-Ulrich Häring, Peter P. Nawroth, Thomas Fleming

**Affiliations:** 1 German Centre for Diabetes Research (DZD), Helmholtz Centre Munich, 85764 Munich, Germany; 2 Institute for Clinical Chemistry and Pathobiochemistry, Department for Diagnostic Laboratory Medicine, University Hospital Tübingen, 72016 Tübingen, Germany; 3 Institute for Diabetes Research and Metabolic Diseases, Helmholtz Centre Munich, University of Tübingen, 72016 Tübingen, Germany; 4 Department of Medicine I and Clinical Chemistry, Heidelberg University Hospital, INF 410, 69120 Heidelberg, Germany; 5 Joint Heidelberg-IDC Translational Diabetes Program, Internal Medicine I, Heidelberg University Hospital, 69120 Heidelberg, Germany; 6 Department of General, Visceral and Transplant Surgery, Eberhard-Karls-University Tübingen, 72016 Tübingen, Germany; 7 Division of Diabetology, Endocrinology and Nephrology, Department of Internal Medicine IV, Eberhard-Karls-University Tübingen, 72016 Tübingen, Germany; 8 Institute for Immunology, University Hospital of Heidelberg, INF 305, 69120 Heidelberg, Germany

**Keywords:** advanced glycation endproducts, dicarbonyls, glyoxalase 1, insulin resistance, liver triglycerides, NAFL

## Abstract

The underlying molecular mechanisms for the development of non-alcoholic fatty liver (NAFL) and its progression to advanced liver diseases remain elusive. Glyoxalase 1 (Glo1) loss, leading to elevated methylglyoxal (MG) and dicarbonyl stress, has been implicated in various diseases, including obesity-related conditions. This study aimed to investigate changes in the glyoxalase system in individuals with non-pathological liver fat. Liver biopsies were obtained from 30 individuals with a narrow range of BMI (24.6–29.8 kg/m^2^). Whole-body insulin sensitivity was assessed using HOMA-IR. Liver biopsies were analyzed for total triglyceride content, Glo1 and Glo2 mRNA, protein expression, and activity. Liquid chromatography–tandem mass spectrometry determined liver dicarbonyl content and oxidation and glycation biomarkers. Liver Glo1 activity showed an inverse correlation with HOMA-IR and liver triglyceride content, but not BMI. Despite reduced Glo1 activity, no associations were found with elevated liver dicarbonyls or glycation markers. A sex dimorphism was observed in Glo1, with females exhibiting significantly lower liver Glo1 protein expression and activity, and higher liver MG-H1 content compared to males. This study demonstrates that increasing liver fat, even within a non-pathological range, is associated with reduced Glo1 activity.

## 1. Introduction

Non-alcoholic fatty liver (NAFL) is not only a potential precursor for the development of non-alcoholic fatty liver diseases (NAFLD) but also represents a strong risk factor for the development of insulin resistance, type-2 diabetes, and cardiovascular events [1,2,3]. Conversely, obesity, insulin resistance, and particularly type-2 diabetes are associated with elevated liver fat. Patients with NAFL are clinically asymptomatic, and NAFL may progress to NAFLD and liver fibrosis, with an elevated risk of cirrhosis and hepatocellular carcinoma [4]. However, the underlying molecular mechanism for the development of NAFL and the subsequent advances to later stages of liver diseases remains unclear [5,6,7,8,9,10].

Advanced glycation endproducts (AGEs) have been reported to be involved in the development of the metabolic syndrome and type-2 diabetes [11,12]. AGEs are a type of post-translational modification resulting from the non-enzymatic reaction of glucose, dicarbonyls, and other saccharide derivatives with the free amino groups of the N-terminal residues, lysine side chains, and guanidino groups of arginine residues within proteins. Methylglyoxal (MG) is an endogenous dicarbonyl resulting from the non-enzymatic degradation of the triosephosphate pool of metabolites in glycolysis [11]. The major AGE formed by MG in vitro and in vivo is the arginine-derived hydroimidazolone, N δ-(5-hydro-5-methyl-4-imidazolon-2-yl)-ornithine (MG-H1). Studies have shown that protein modification by methylglyoxal (MG), leading to the formation of arginine-derived hydroimidazolone (MG-H1), results in structural changes and functional impairment [13,14,15]. Free MG-H1, released from modified proteins by cellular proteolysis, has been detected in plasma. Elevated levels of MG-H1 have been associated with various diseases, including diabetes [11].

The glyoxalase system is the primary pathway responsible for detoxifying methylglyoxal (MG) and mitigating MG-induced glycation, known as dicarbonyl stress [11]. It is a highly conserved system expressed ubiquitously in the cytoplasm of mammalian cells. The system comprises two enzymes: glyoxalase I (Glo1) and glyoxalase II (Glo2), along with a catalytic amount of reduced glutathione (GSH). Glo1 catalyzes the conversion of a hemithioacetal adduct, spontaneously formed between MG and GSH, to S-D-lactoylglutathione, which is further hydrolyzed to D-lactate by Glo2 [16,17]. Animal studies have revealed decreased Glo1 activity in the liver in response to both fructose and high-fat diets [18,19,20]. Conversely, overexpression of Glo1 in high-fat diet mice suppressed body weight gain and adiposity, with similar food consumption compared to wild-type high-fat fed mice [21]. Increased advanced glycation endproducts (AGEs) in the liver have been associated with the severity of steatosis in individuals with non-alcoholic fatty liver disease NAFLD [22]. The induction of Glo1 in overweight and obese subjects through the dietary supplement combination of trans-resveratrol and hesperetin has been reported to counter the accumulation of methylglyoxal, while also reversing insulin resistance and improving dysglycemia and low-grade inflammation [23,24]. In skeletal muscle of obese, type-2 diabetic individuals, Glo1 protein expression was significantly reduced and correlated with body mass index (BMI), percentage of body fat, and HOMA-IR [25]. Analysis of liver biopsies and plasma from individuals with NAFLD showed no relationship between plasma MG-H1, inflammation, or NAFLD activity score, but a significant correlation with BMI [20]. Liver MG-H1 was also found to be elevated in obese individuals with histologically proven NASH without type-2 diabetes, compared to healthy individuals without liver disease or obese individuals with type-2 diabetes [26]. These findings suggest that the downregulation of Glo1, leading to an increase in MG and dicarbonyl stress, may contribute to the pathogenesis of NAFLD [27,28]. Although the pathogenesis of NAFLD has been extensively studied, less is known about preclinical dysregulations in livers with non-pathological fat accumulation.

The aim of this study was to investigate the presence of changes in the glyoxalase system in individuals with non-pathological liver fat. To achieve this objective, intraoperative liver biopsies from metabolically characterized individuals with varying degrees of liver fat were biochemically assessed in terms of the glyoxalase system.

## 2. Materials and Methods

### 2.1. Subjects

Caucasian individuals (5 women/25 men); age, 64.5 (56–71.25) years; body mass index, (26.6, 24.6–29.8) kg/m^2^; median and interquartile range) undergoing liver surgery (e.g., for the resection of solitary hepatic lesions) were included in the present study (Department of General, Visceral and Transplant Surgery at the University of Tübingen). Patients fasted overnight before collection of the liver biopsies and corresponding blood samples. Subjects tested negative for viral hepatitis and had no liver cirrhosis and were rated normal by a pathologist. Liver samples were taken from normal, non-diseased tissue determined by the pathologist during surgery, immediately frozen in liquid nitrogen and stored at −80 °C.

### 2.2. Determination of Liver Triglyceride

The triglyceride content in the liver tissue was determined as previously described [29].

### 2.3. Clinical Chemistry Parameters

Creatinine, plasma glucose, alanine aminotransferase, aspartate aminotransferase, C-reactive protein, total, HDL, and LDL cholesterol) were determined on an ADVIA XPT Clinical Chemistry system (Siemens Healthineers, Erlangen, Germany). Plasma insulin was determined on the ADVIA Centaur XPT chemiluminometric immunoassay system (Siemens Healthineers). Glycated hemoglobin (HbA1c) was determined using the Tosoh G8 HPLC analyzer (Tosoh Bioscience, Griesheim, Germany).

### 2.4. Measurement of Dicarbonyls

The dicarbonyl content in the liver tissue was determined by isotope dilution and tandem mass spectroscopy, following derivatization with 1,2-diaminobenzene, as previously described [30].

### 2.5. Measurement of Protein-Bound Glycation and Oxidation Biomarkers

Protein-bound glycation (MG-H1, CML, CEL, MOLD and fructosyl-lysine) and oxidation (methionine sulphoxide and dityrosine) biomarkers in the liver were determined by isotope dilution and tandem mass spectroscopy, as previously described [31].

### 2.6. Real-Time PCR

For RNA isolation and quantitative real-time (RT)-PCR analysis of hepatic mRNA expression, frozen tissue was homogenized in a TissueLyser (Qiagen, Hilden, Germany), and RNA was extracted with the RNeasy Tissue Kit (Qiagen) according to the manufacturer’s instructions. Total RNA treated with RNase-free DNase I was transcribed into cDNA by using a first-strand cDNA kit, and PCRs were performed in duplicates on a LightCycler480 (Roche Diagnostics, Mannheim, Germany). The primers used were as follows: Glo1: 5′-TCCCGTCGTCTGTGATACTG-3′, 5′-ACTCGTAGCATGGTCTGCTG-3; Glo2: 5′-TGGCGGGAATGAGAAACTGG-3′, 5′-TTGACGTTCAGAGACCCCAC-3′ and Rps13: 5′-CCCCACTTGGTTGAAGTTGA-3′, 5′-ACACCATGTGAATCTCTCAGGA-3′. Data are presented relative to the housekeeping gene (Rps13) using the ΔΔCt method.

### 2.7. Western Blotting

Total protein extracts from the liver (ca. 10 mg) were prepared by homogenization in 10 mM sodium phosphate buffer (pH 7.4) containing protease inhibitor cocktail. The soluble protein fraction was retained following centrifugation (14,000 rpm; 5 min; 4 °C) and protein concentration was determined by Bradford assay [32]. A total of 10µg of protein was incubated with 1 × Laemmli buffer at 95 °C for 10 min and separated by a Mini-PROTEAN^®^ TGX (Bio-Rad, Hercules, CA, USA) pre-casted gel (4–20% acrylamide). Proteins were then transferred to a nitrocellulose membrane and blocked with 2% dry milk (in PBS) at room temperature for 1 h. Membranes were then incubated overnight at 4 °C with antibodies against Glo1 (1:1000; Abcam, Cambridge, UK, ab81461; rat), Glo2 (1:1000; Abcam, ab154108; rabbit) or ß-actin (1:2000; Cell Signaling, Danvers, MA, USA, 4967S; rabbit) in 2% dry milk containing PBS and 0.05% Tween20 (PBS-T). After three washing steps (5 min each) with PBS-T, membranes were incubated with appropriate horseradish-linked secondary antibody for 1 h at room temperature. Proteins were then visualized on X-ray films using ECL detection reagents (GE healthcare, Chicago, IL, USA) with varying exposure time (0.1–2 min). The full blots are shown in Supplementary Figure S1.

### 2.8. Measurement of Glo1 Activity

The activity of Glo1 was determined spectrophotometrically by measuring the initial rate of increase in absorbance at 235 nm following addition of total protein extract (ca. 10 µg protein) to hemithioacetal, prepared by pre-incubating 2 mM methylglyoxal and 2 mM GSH in 50 mM sodium phosphate buffer (pH 6.6). For the conversion of the hemithioacetal to S-D-lactoylglutathione, the change in molar extinction coefficient Δε235 = 1.07 mM^−1^cm^−1^ was used. The activity of Glo1 was expressed in units (U), where 1 U is the amount of Glo1 which catalyzes the formation of 1µmol of S-D-lactoylglutathione per minute at 37 °C [33].

### 2.9. Measurement of Glo2 Activity

The activity of Glo2 was determined spectrophotometrically by measuring the initial rate of formation of reduced glutathione from S-D-lactoylglutathione following the addition of total protein extract (ca. 10 µg protein). Reduced glutathione is measured by reaction with 5′5-dithiobis(2-nitrobenzoic) acid (DNTB) at 415 nm. The reaction buffer contained 0.3 mM S-D-lactoylglutathione and 0.16 mM DNTB in 50 mM Tris-HCl (pH 7.4). The amount of GSH was determined with reference to a calibration curve containing known amounts of GSH (50–2000 pmol). The activity of Glo2 was expressed in units (U), where 1 U is the amount of Glo2 which catalyzes the formation of 1 µmol of GSH per minute at 37 °C [34].

### 2.10. Statistical Analyses and Calculations

Whole-body insulin sensitivity was measured using the homoeostatic measurement assessment of insulin resistance (HOMA-IR) [35]. BMI was calculated as weight divided by height squared (kg/m^2^). Statistical data analysis was performed using GraphPad Prism 7 (GraphPad Software Inc., San Diego, CA, USA). Data that were not normally distributed (Shapiro–Wilk W-test) and logarithmically transformed. Linear regression and Pearson correlation coefficient were used to study associations between measured variables. For testing between two data sets, unpaired, Mann–Whitney U test. Differences were considered significant at *p* < 0.05

## 3. Results

**Figure 1:**
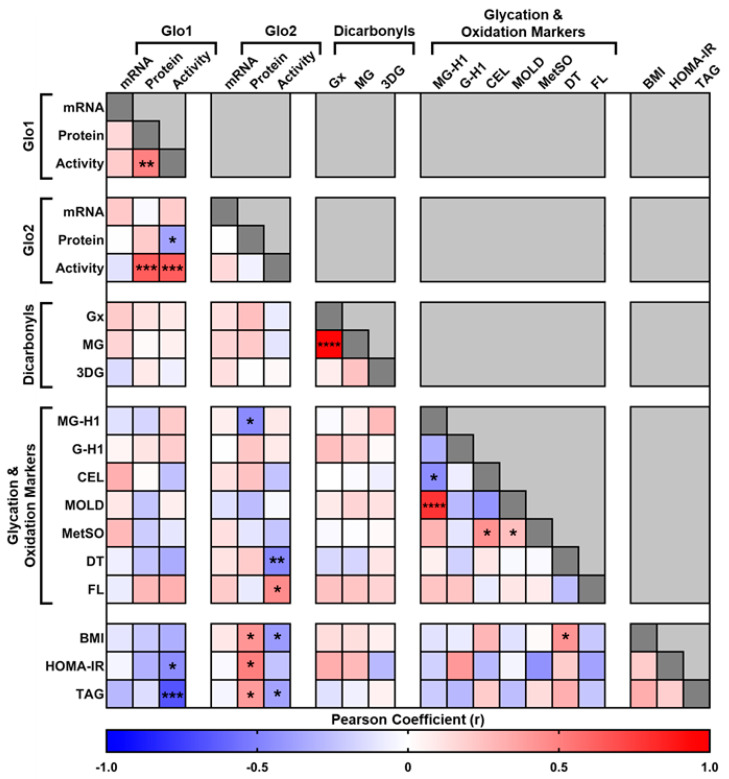
Correction matrix of glyoxalase system and clinical variables in liver biopsies obtained from metabolically characterized patients. Glo1 = Glyoxalase 1, Glo2 = Glyoxalase 2, mRNA = Messenger RNA, Gx = Glyoxal, MG = Methylglyoxal, 3DG = 3−Deoxyglucosone, MG−H1 = Methylglyoxal−derived hydroimidazolone isomer−1, G−H1 = Glyoxal−derived hydroimidazolone isomer−1, CEL = Nε−(1-Carboxyethyl)−L−lysine, MOLD = Methylglyoxal−derived lysine dimer, MetSO = Methionine Sulphoxide, DT = Dityrosine, FL = Fructosyl−lysine, BMI = Body Mass Index, HOMA−IR = Homeostatic model assessment for insulin resistance, TAG = Triglycerides. **** *p* < 0.0001, *** *p* < 0.001, ** *p* < 0.01 and * *p* < 0.05. Unless specified all other correlations were non-significant (*p* > 0.05).

**Figure 2:**
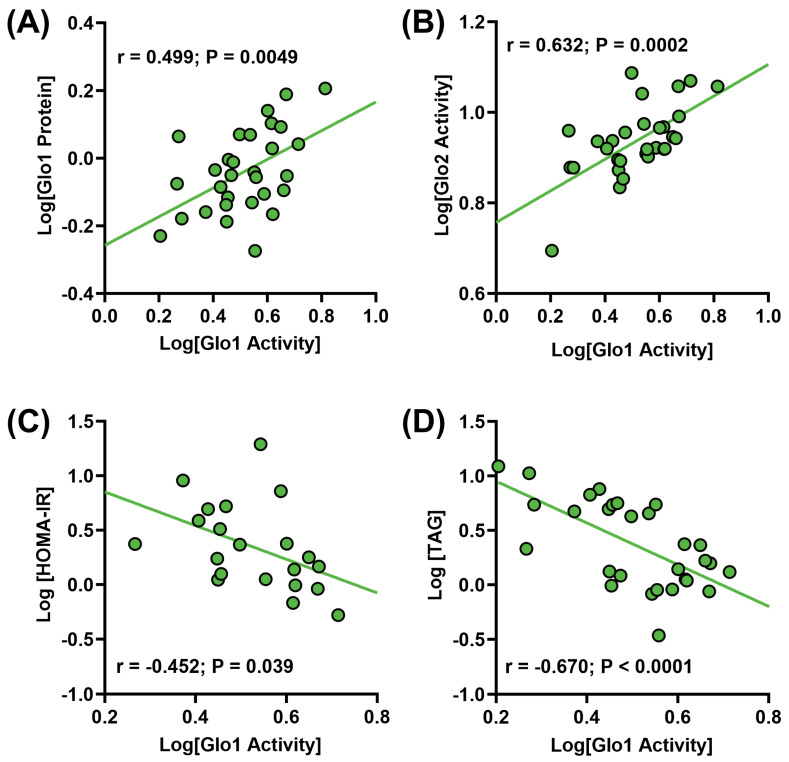
Correlation analysis observed between (**A**) liver glyoxalase I (Glo1) activity and protein expression; (**B**) liver Glo1 activity and liver glyoxalase II (Glo2) activity; (**C**) liver Glo1 activity and HOMA−IR; and (**D**) liver Glo1 activity and liver triglycerides (TAG).

In order to establish significant correlations between the clinical variables and the measurements of the glyoxalase system, as well as glycation and oxidation protein damage biomarkers (Table 1), a correlation matrix was constructed based upon the normalized data (Figure 1). The glyoxalase system is comprised of two synergistic enzymes, Glo1 and Glo2. There were no associations between the mRNA levels of either Glo1 or Glo2 and the respective protein levels and/or activity, consistent with the findings that mRNA expression does not directly predict protein expression [36,37]. There was a significant, positive correlation between Glo1 protein expression and activity (Figure 1 and Figure 2A), which would be expected given that activity of an enzyme is closely related to its abundance within the proteome.

There was no correlation, however, between the protein expression and activity of Glo2, suggesting that the activity of this enzyme is not directly related to its expression. It was subsequently found that the activity of Glo2 significantly correlated with Glo1 protein expression (r = 0.636; r^2^ = 0.405; *p* = 0.0002), as well as activity (r = 0.632; r^2^ = 0.400; *p* = 0.0002; Figure 1 and Figure 2B).

The protein expression of Glo2 was not associated with the protein expression of Glo1, but was negatively associated with the activity of Glo1 (r = −0.368; r^2^ = 0.135; *p* = 0.045; Figure 1), suggesting that the activity of Glo2 is dependent upon the activity of Glo1, consistent with the synergistic nature of the reaction between the two enzymes and that Glo1 is the rate-limiting enzyme in the glyoxalase system. There were no associations between Glo1 protein expression and BMI, HOMA-IR or liver triglyceride content.

However, the activity of Glo1 was found to be negatively correlated with HOMA-IR (Figure 1 and Figure 2C) and liver triglycerides (Figure 1 and Figure 2D). There was also a negative correlation with BMI; however, it was not significant (r = −0.321; r^2^ = 0.103; *p* = 0.083). In contrast, the protein expression of Glo2 was found to correlate positively with BMI (r = 0.418, r^2^ = 0.175, *p* = 0.021), HOMA-IR (r = 0.503; r^2^ = 0.253; *p* = 0.02) and liver triglycerides (r = 0.395; r^2^ = 0.156; *p* = 0.030) (Figure 1). The activity of Glo2 was not associated with HOMA-IR, but did correlate negatively with BMI (r = −0.403, r^2^ = 0.163, *p* = 0.026) and liver triglycerides (r = −0.366; r^2^ = 0.134; *p* = 0.046) (Figure 1).

The glyoxalase system is the major pathway for the detoxification of dicarbonyls, in particular, MG. Despite this functional link, there were no significant associations between the liver dicarbonyl content and the glyoxalase system (Figure 1). The only association observed was between the liver content of MG and glyoxal, which significantly correlated to each other (r = 0.948, r^2^ = 0.899, *p* < 0.0001). There were also no associations between the liver dicarbonyls and BMI, HOMA-IR, or liver triglyceride content (Figure 1). Dicarbonyls are also potent glycating agents leading to the formation of stable AGEs. There were also no associations of the liver dicarbonyls and the protein damage markers of glycation and oxidation. There were, however, significant associations between the different markers. For instance, the MG-derived AGEs, MG-H1, and MOLD were found to significantly correlate to each other (r = 0.786, r^2^ = 0.617, *p* < 0.0001), whilst CEL negatively correlated to MG-H1 (r = −0.454, r^2^ = 0.206, *p* = 0.011). The oxidation marker, methionine sulphoxide, was found to positively correlate with CEL (r = 0.432, r^2^ = 0.186, *p* = 0.017) and MOLD (r = 0.251, r^2^ = 0.063, *p* = 0.020; Figure 1). With respect to the glyoxalase system, there were no associations between the glycation and oxidation protein damage biomarkers and Glo1. However, liver MG-H1 was found to negatively correlate to the protein expression of Glo2 (r = −0.460, r^2^ = 0.212, *p* = 0.010). The early glycation adduct, fructosyl-lysine, was found to positively correlate with Glo2 activity (r = 0.451, r^2^ = 0.204, *p* = 0.012), whilst the oxidation marker, dityrosine, negatively correlated to the activity of Glo2 (r = −0.469, r^2^ = 0.220, *p* = 0.008; Figure 1). There were no associations between the liver glycation and oxidation protein damage markers and BMI, HOMA-IR, or liver triglyceride content. However, the oxidation marker, dityrosine, was found to correlate significantly with BMI (r = 0.422, r^2^ = 0.178, *p* = 0.019), but not with either HOMA-IR or liver triglyceride content (Figure 1).

**Figure 3:**
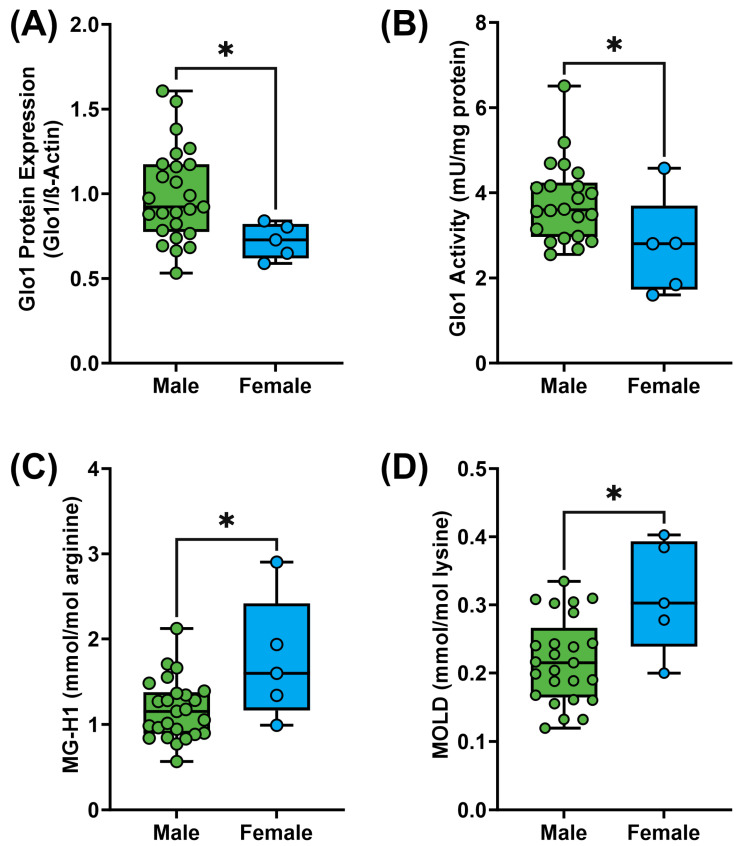
Sex dimorphism with respect to liver Glo1 protein expression (**A**), activity (**B**), MG-H1 (**C**), and MOLD (**D**) content. Data are shown as min-to-max box plots, with the median indicated by the line in the middle of the box; * *p* < 0.01.

Regardless of the BMI, HOMA-IR, or liver triglyceride content, a sex dimorphism was observed with respect to the Glo1 (Supplementary Table S1). The protein expression of Glo1 was found to be significantly decreased in females, as compared to males (0.994 ± 0.276 vs. 0.722 ± 0.105 A.U.; *p* = 0.02; Figure 3A). This was paralleled by a similar significant decrease in Glo1 activity (3.79 ± 0.94 vs. 2.73 ± 1.17 mU/mg; *p* = 0.04; Figure 3B). There was no significant difference in liver methylglyoxal between males and females (Supplementary Table S1); however, there was a significant increase in liver MG-H1 (1.17 ± 0.35 vs. 1.75 ± 0.73 mmol/mol arginine; *p* = 0.05; Figure 3C) and MOLD (0.22 ± 0.06 vs. 0.31 ± 0.08 mmol/mol lysine; *p* = 0.03; Figure 3D).

## 4. Discussion

Glo1 has been implicated in various diseases, including obesity-related conditions. Previous studies suggest that the downregulation of Glo1, resulting in increased MG levels and dicarbonyl stress, may contribute to the pathogenesis of non-alcoholic fatty liver NAFL [26,27]. For example, elevated advanced glycation endproducts (AGEs) in the liver have been linked to the severity of steatosis in individuals with non-alcoholic fatty liver disease (NAFLD) [22]. Liver MG-H1 levels were also higher in obese individuals with histologically proven NASH but without type-2 diabetes, compared to healthy individuals without liver disease or obese individuals with type-2 diabetes [26]. In the analysis of liver biopsies and plasma from individuals with NAFLD, no significant relationship was found between plasma MG-H1, inflammation, or NAFLD activity score, but a significant correlation was observed with BMI [20]. However, in the same study, no differences in Glo1 expression, assessed by immunohistochemistry, were noted in the livers of individuals with NAFLD. Nevertheless, differences in the subcellular localization of Glo1 were observed; staining was cytoplasmic in non-NAFLD controls and predominantly nuclear in individuals with NAFLD [20].

The aim of this study was to determine whether changes previously described in the glyoxalase system, concerning liver injury, were evident in individuals with non-pathological liver fat accumulation. To achieve this goal, intraoperative liver biopsies were obtained from metabolically characterized individuals (BMI 24.6–29.8; 25–75% percentile) with varying degrees of liver fat. These biopsies were then biochemically assessed for glyoxalase system activity. The study revealed a significant, inverse correlation between liver Glo1 activity and HOMA-IR, and to a greater extent, liver triglycerides. This suggests that the glyoxalase system may be downregulated in fatty liver disease, consistent with previous in vivo findings [20]. Interestingly, no correlation was found between BMI, HOMA-IR, and liver triglycerides, indicating that in non-pathological conditions, these parameters may not be directly associated. It suggests that the level of obesity and liver damage may not yet be sufficient to affect whole-body insulin sensitivity. Therefore, Glo1 activity could serve as a prognostic marker for the early detection of fatty liver disease.

The loss of Glo1 activity was not associated with a change in protein expression, suggesting that the decline in activity may be due to detrimental post-translational modifications (PTMs), as previously reported [38,39,40,41,42,43]. Dityrosine (DT) was found to be the only PTM significantly positively correlated with BMI, indicating enhanced oxidative stress. Interestingly, methionine sulphoxide, another marker for oxidative stress, did not show the same association, likely due to the actions of the methionine sulphoxide reductase superfamily, which can reduce methionine sulphoxide to methionine [44]. Studies have shown that in obesity and early obesity-related NAFL, hepatic mitochondrial respiration is upregulated as an adaptive response to adipose tissue-derived lipid flux to the liver, associated with increased production of reactive oxygen species (ROS) and lipid peroxidation [45]. In skeletal muscle from obese type-2 diabetic individuals, the loss of Glo1 protein expression was associated with an increase in global carbonyl stress, covering both oxidative and carbonyl modifications, rather than MG-derived modifications [25]. This suggests that mitochondrial-driven oxidative stress underlies the loss of Glo1. However, as there was no correlation between Glo1 protein and/or activity and DT, it suggests that the potential mechanism is an indirect consequence of enhanced oxidative stress, rather than the result of direct oxidative modification of Glo1. For instance, increased oxidative stress can affect the redox balance within the cell, leading to activation of hypoxia-inducible factor-1a (HIF1a), which has been shown to downregulate Glo1 expression [46] or the glutathionylation of Glo1 and the loss of activity [39].

No association was found between Glo2 protein expression and activity, suggesting that activity is not dependent on its protein expression. Glo2 activity could also be regulated by PTMs. However, while there is evidence to suggest that Glo2 is involved in the regulation of several PTM [47], there is no evidence to suggest that Glo2 is a target for functionally relevant modifications. Subsequently, it was found that Glo2 activity was more strongly associated with the protein expression and/or activity of Glo1. This is consistent with the synergistic nature of the reactions between the two enzymes and the fact that Glo1 is the rate-limiting enzyme in the glyoxalase system [11,16]. The inverse correlation of Glo2 activity with liver triglycerides may therefore simply reflect the changes observed in Glo1 protein expression and activity.

The reduction in liver Glo1 activity was not associated with increases in either liver MG or MG-H1. Similar findings have been reported in the skeletal muscle of obese, insulin-resistant individuals. However, this reduction was only observed in Glo1 protein expression and not activity, which was not determined; neither was an increase in MG-H1 modified proteins [25]. The lack of any associations between the glyoxalase system and MG/MG-H1 could be due to the dynamic production of MG, which is closely linked to glycolytic rate and the accumulation of triosephosphate intermediates [11]. The measurements performed in this study represent the steady-state metabolism within the liver and may not necessarily reflect the potential for increased production, which could only be determined from metabolic flux analysis (MFA). Additionally, the lack of an increase could also be attributed to the activity of aldo-keto reductase, which has been shown to be an effective means for MG detoxification in the absence of Glo1 [48,49]. Unfortunately, due to the limited amount of metabolically well-characterized human material available, the activity of aldo-keto reductase and the determination of alternative detoxification pathways could not be performed in this cohort. Further studies are therefore required to determine the relative contribution of aldo-keto reductase and alternative detoxification pathways with respect to changes observed in Glo1 activity.

In this study, a sex dimorphism independent of liver triglycerides was observed regarding Glo1. Females exhibited lower protein expression and activity of liver Glo1, along with higher levels of liver MG-H1. This suggests that females may be more susceptible to liver diseases involving the accumulation of MG/MG-H1. This finding is counterintuitive, considering that the literature indicates that females are less susceptible to metabolic disorders and liver disease due to factors such as reduced expression of nuclear receptors like PPARα, increased activation of glucocorticoid receptors, and increased expression of xenobiotic receptors allowing for enhanced detoxification of xenobiotic compounds compared to males [50,51]. Additionally, the opposite trend in Glo1 activity/protein has been reported in atherosclerotic carotid artery lesions [52]. However, females were underrepresented in this study compared to males. Further research is needed to validate the observed Glo1 sex dimorphism and its implications. Moreover, larger studies including a more diverse cohort with a wider range of BMI and liver triglycerides, representative of NAFL and NASH, are required to better define the pathologically relevant associations observed regarding the glyoxalase system.

This study has several limitations that warrant consideration. Based on the design of our human study using intraoperative liver biopsies of metabolically well-characterized patients there are some intrinsic limitations. Firstly, the sample size and composition of the study cohort may limit the generalizability of our findings. Secondly, according to our study design, the patients cannot be selected according to their age, gender, metabolic status, and other parameters. Thus, the results of our study may not fully represent the broader population with diverse metabolic characteristics and varying degrees of liver fat accumulation. Additionally, the cross-sectional design of the study restricts our ability to establish causality or assess changes over time. However, longitudinal studies would require repeated/sequential sampling, a practice which was not covered by the approved ethics protocol of this study. Lastly, the findings of this study may not be applicable to populations with different genetic backgrounds, lifestyles, ethnicities, or environmental exposures.

In conclusion, this study identifies the role of Glo1 activity in liver health, particularly in relation to the accumulation of fat. The observed inverse correlation between liver Glo1 activity and markers of insulin resistance and liver triglycerides suggests a potential prognostic value of Glo1 activity in early fatty liver disease detection. Interestingly, the decrease in Glo1 activity was not accompanied by significant changes in liver MG or MG-H1 levels, highlighting the complexity of glyoxalase system regulation in non-pathological liver fat accumulation. These findings emphasize the need for further research to validate these observations and uncover the underlying pathophysiological mechanisms governing the role of Glo1 in liver health and disease.

## Figures and Tables

**Table 1:** Anthropometric characteristics, biochemical assessment of the liver glyoxalase system, liver dicarbonyls, and glycation and oxidation biomarkers of the study cohort.

Parameter	Median (25–75 Percentile)
Gender	30 (25 M/5 F)
Age (years)	64.5 (56–71.25)
Weight (kg)	81.5 (76.5–91)
Height (m)	175 (169–180)
BMI (kg/m^2^)	26.6 (24.6–29.8)
Liver fat (%)	1.9 (1.1–5.4)
HOMA-IR (μU/mL × mmol/L; n = 21 (18 M/3 F))	1.78 (1.11–4.39)
**Liver Glyoxalase System**	
Glo1 mRNA (Rps13 mRNA Normalized)	0.412 (0.319–0.490)
Glo1 Protein Expression (Actin Normalized)	0.889 (0.736–1.163)
Glo1 Activity (mU/mg)	3.46 (2.77–4.15)
Glo2 mRNA (Rps13 mRNA Normalized)	0.047 (0.040–0.058)
Glo2 Protein Expression (Actin Normalized)	1.08 (0.98–1.30)
Glo2 Activity (mU/mg)	8.49 (7.84–9.32)
**Liver Dicarbonyls (pmol/mg)**	
Glyoxal	7.79 (4.81–9.85)
Methylglyoxal	4.50 (3.58–6.18)
3DG	0.299 (0.269–0.418)
**Liver Glycation Biomarkers**	
MG-H1 (mmol/mol Arg)	1.22 (0.93–1.50)
G-H1 (mmol/mol Arg)	0.245 (0.131–0.308)
CEL (mmol/mol Lys)	1.004 (0.253–1.810)
MOLD (mmol/mol Lys)	0.222 (0.183–0.302)
Fructosyl-lysine (mmol/mol Lys)	9.995 (8.583–12.841)
**Liver Oxidation Biomarkers**	
Methionine Sulphoxide (mmol/mol Met)	32.17 (20.88–42.87)
Dityrosine (mmol/mol Tyr)	0.07 (0.040–0.105)

## Data Availability

Data will be made available on request due to privacy.

## Supplementary Materials

The following supporting information can be downloaded at: https://www.mdpi.com/article/10.3390/metabo14040209/s1, Supplementary Table S1. Sex differences in anthropometric characteristics, biochemical assessment of the liver glyoxalase system, liver dicarbonyls and glycation and oxidation biomarkers of the study cohort. Supplementary Figure S1. (A) Full blot for Glyoxalase 1 (Glo1) protein analysis. Region taken for densitometer analysis are shown in red. (B) Full blot for Actin protein analysis. Region taken for densitometer analysis are shown in red. (C) Full blot for Glyoxalase 2 (Glo2) protein analysis. Region taken for densitometer analysis are shown in red. (D) Full blot for Actin protein analysis. Region taken for densitometer analysis are shown in red. (E) Summary of Western blot analysis for Glo1, Glo2, and Actin.
