# Supplementary material for: Accumulation of Non-Pathological Liver Fat Is Associated with the Loss of Glyoxalase I Activity in Humans
Source: Metabolites. 2024 Apr 7;14(4):209. doi: 10.3390/metabo14040209 (PMC11051733; doi:10.3390/metabo14040209)

Supplementary Table(s):

Supplementary Table 1. Sex differences in anthropometric characteristics, biochemical assessment of the liver glyoxalase system, liver dicarbonyls and glycation and oxidation biomarkers of the study cohort.

**Parameter**	**Male**	**Female**	**P-value**
No. of Individuals	25	5	<0.001
Age (Years)	63 ± 11	62 ± 3	0.33
BMI (kg/m2)	26.8 ± 3.19	27.8 ± 4.49	0.83
TAG (% of Liver Tissue)	3.12 ± 2.72	4.46 ± 4.58	0.36
HOMA-IR	3.77 ± 4.59	1.73 ± 0.624	0.67
**Liver Glyoxalase System**			
Glo1 mRNA (Rps13 mRNA Normalized)	0.42 ± 0.14	0.36 ± 0.05	0.21
Glo1 Protein Expression (Actin Normalized)	0.99 ± 0.28	0.72 ± 0.10	0.02
Glo1 Activity (mU/mg)	3.79 ± 0.94	2.73 ± 1.17	0.04
Glo2 mRNA (Rps13 mRNA Normalized)	0.05 ± 0.02	0.05 ± 0.01	0.91
Glo2 Protein Expression (Actin Normalized)	1.13 ± 0.22	1.09 ± 0.25	0.87
Glo2 Activity (mU/mg)	9.01 ± 1.48	7.63 ± 1.64	0.17
**Liver Dicarbonyls (pmol/mg)**			
Glyoxal	8.09 ± 4.76	9.43 ± 6.04	0.59
Methylglyoxal	5.16 ± 2.53	6.10 ± 3.22	0.48
3DG	0.33 ± 0.11	0.37 ± 0.08	0.36
**Liver Glycation Biomarkers**			
MG-H1 (mmol/mol Arg)	1.17 ± 0.35	1.75 ± 0.73	0.05
G-H1 (mmol/mol Arg)	0.25 ± 0.14	0.25 ± 0.13	0.83
CEL (mmol/mol Lys)	1.36 ± 1.72	1.20 ± 0.80	0.87
MOLD (mmol/mol Lys)	0.22 ± 0.06	0.31 ± 0.08	0.03
Fructosyl-lysine (mmol/mol Lys)	10.1 ± 2.88	14.5 ± 7.84	0.15
**Liver Oxidation Biomarkers**			
Methionine Sulphoxide (mmol/mol Met)	31.7 ± 16.7	55.1 ± 22.0	0.009
Dityrosine (mmol/mol Tyr)	0.09 ± 0.06	0.13 ± 0.14	>0.99

Supplementary Figure 1A. Full blot for Glyoxalase 1 (Glo1) Protein Analysis. Region taken for densitometer analysis are shown in red.

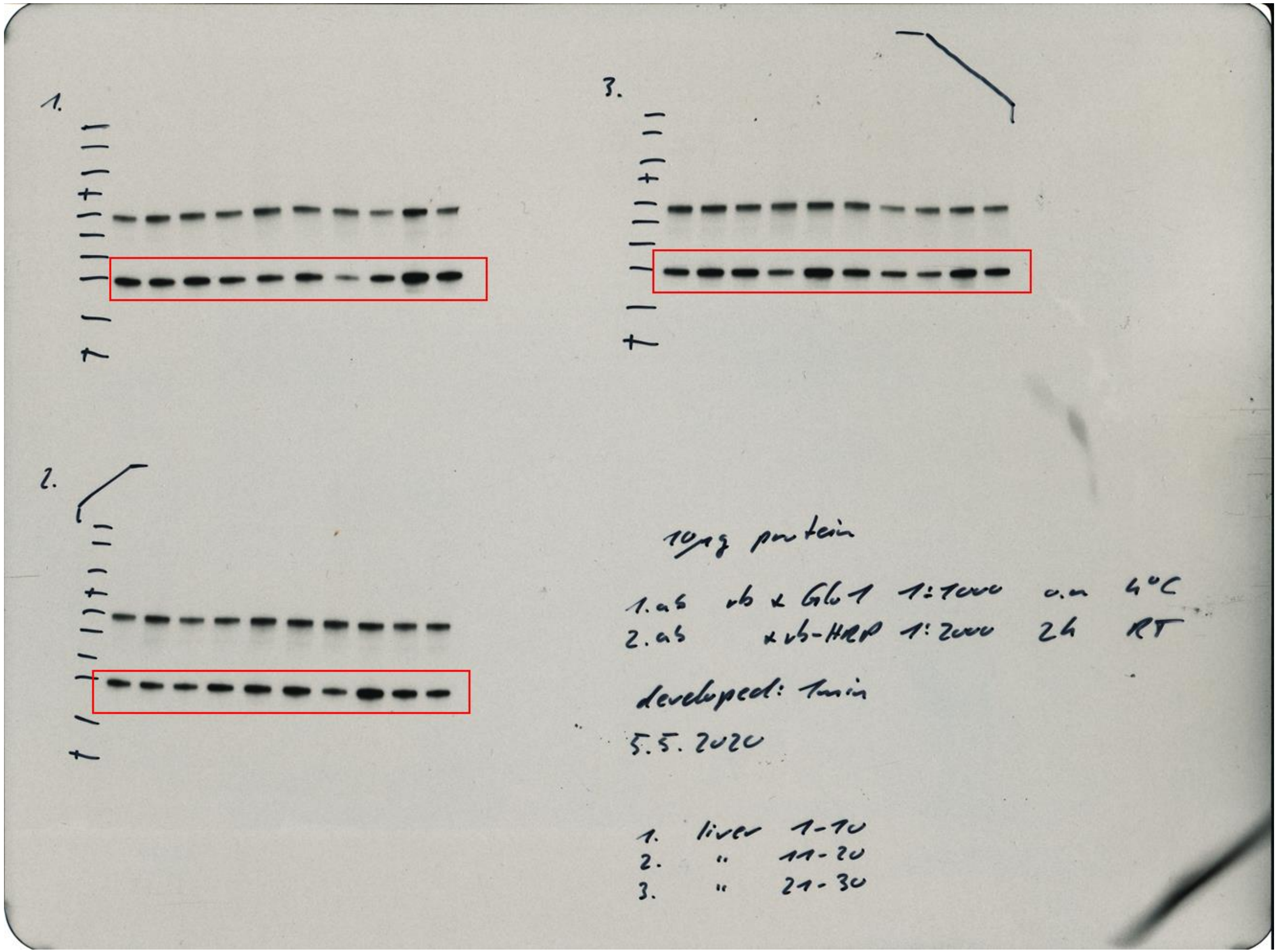

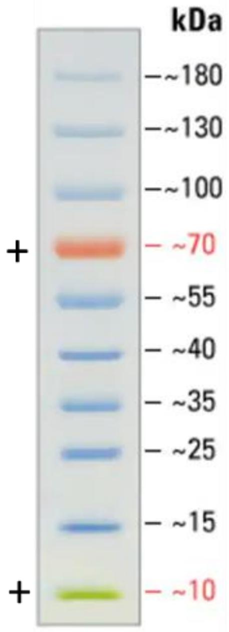

PageRuler™ Prestained Protein Ladder, 10 to 180 kDa (Thermo; 26616)

Supplementary Figure 1B. Full blot for Actin Protein Analysis. Region taken for densitometer analysis are shown in red.

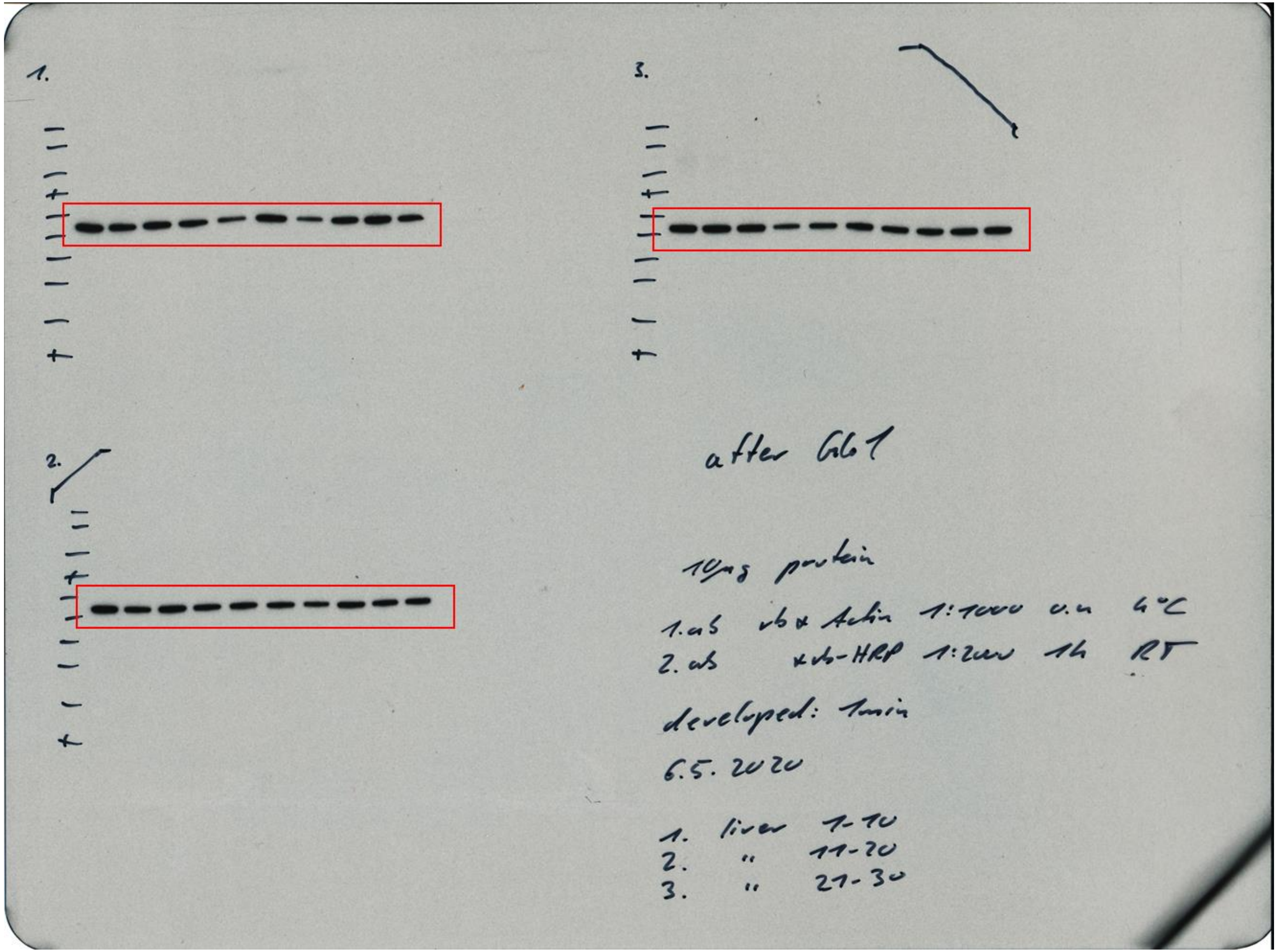

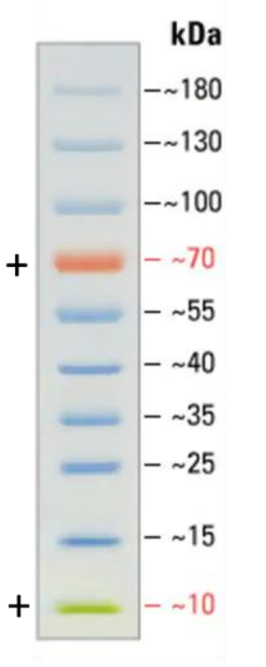

PageRuler™ Prestained Protein Ladder, 10 to 180 kDa (Thermo; 26616)

Supplementary Figure 1C. Full blot for Glyoxalase 2 (Glo2) Protein Analysis. Region taken for densitometer analysis are shown in red.

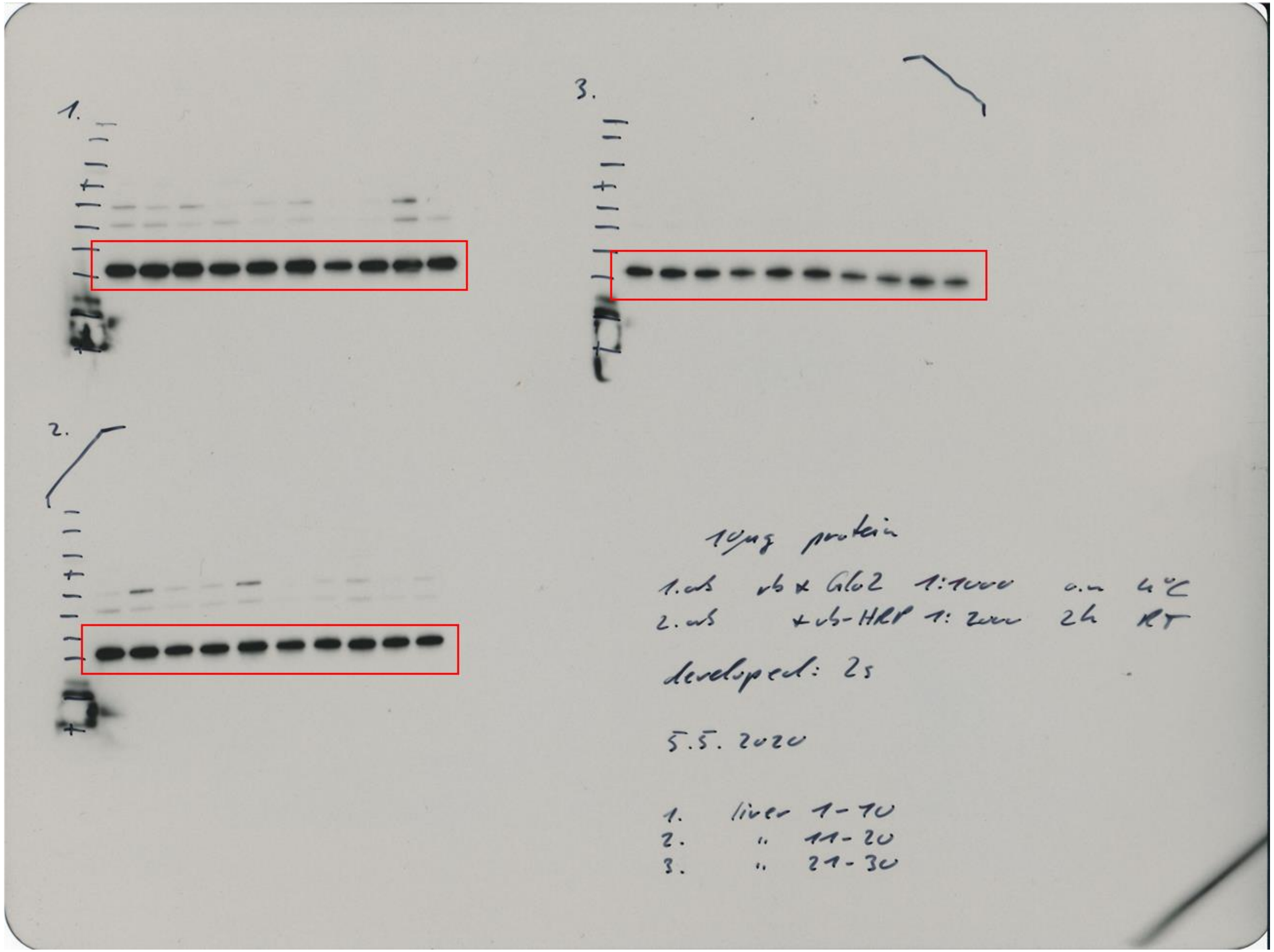

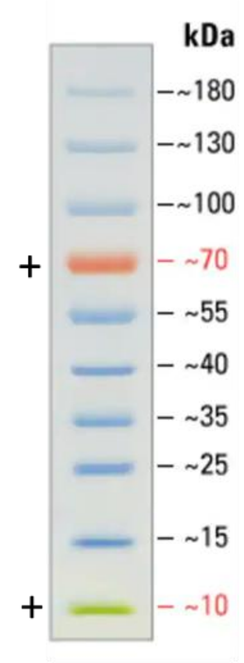

PageRuler™ Prestained Protein Ladder, 10 to 180 kDa (Thermo; 26616)

Supplementary Figure 1D. Full blot for Actin Protein Analysis. Region taken for densitometer analysis are shown in red.

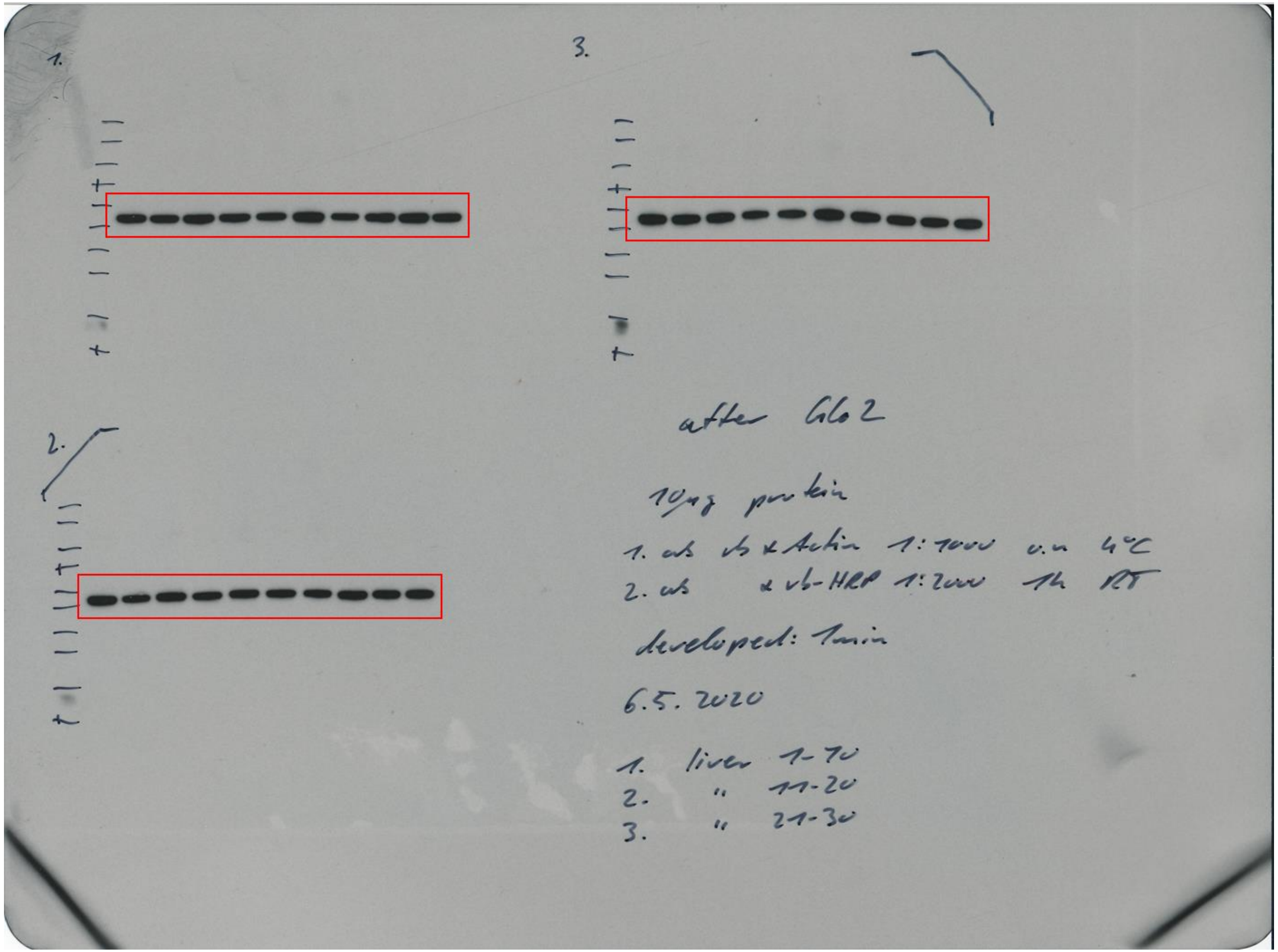

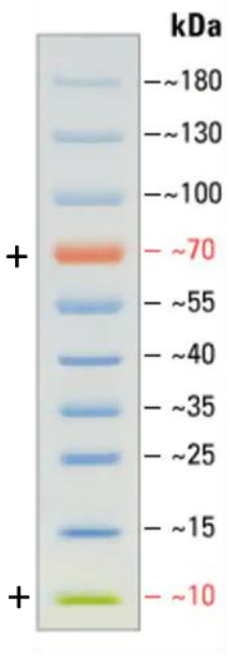

PageRuler™ Prestained Protein Ladder, 10 to 180 kDa (Thermo; 26616)

Supplementary Figure 1E. Summary of Western Blot Analysis for Glo1, Glo2 & Actin.

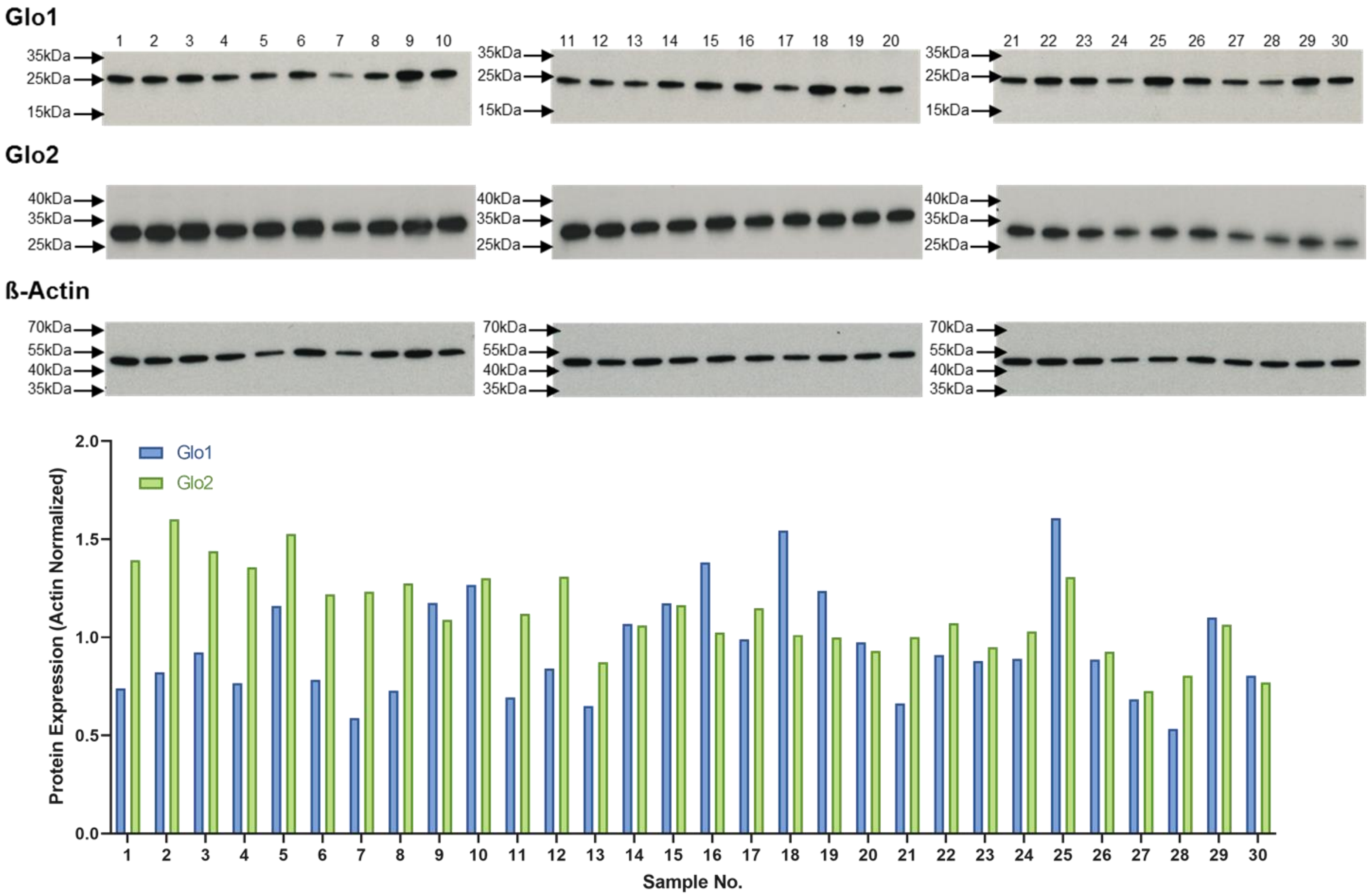

Supplement: Supplementary file 1 [file metabolites-14-00209-s001.zip › metabolites-2928000-supplementary.pdf]
